# Shenze shugan capsules alleviate alcoholic liver injury in association with modulation of the TGF-β/smad4 signaling pathway

**DOI:** 10.3389/fphar.2026.1942947

**Published:** 2026-10-02

**Authors:** Lu Wang, Qianyu Gu, Jia Tinghe, Zhen Sun, Di Wang, Lanzhou Li, Huiyun Zang, Lijun Lin, Junliang Chen, Chunyue Wang

**Affiliations:** 1 Engineering Research Center of Chinese Ministry of Education for Edible and Medicinal Fungi, Jilin Agricultural University, Changchun, China; 2 School of Life Sciences, Jilin University, Changchun, China; 3 Jilin Aodong Shihang Pharmaceutical Co., Ltd., Dunhua, China; 4 Jilin Aodong Pharmaceutical Group Yanji Co., Ltd., Yanji, China; 5 Science and Research Center for Edible Fungi of Qingyuan County, Qingyuan, China

**Keywords:** alcohol-associated liver disease, fibrosis, gut microbiota, SZSG, TGF-β/Smad4

## Abstract

**Background:**

Alcohol-associated liver disease (ALD) represents a major contributor to liver fibrosis globally, and current therapeutic options remain inadequate. The TGF-β/Smad4 cascade is critically involved in alcohol-evoked activation of hepatic stellate cells and subsequent extracellular matrix accumulation. Although Shenze Shugan capsule (SZSG) has been clinically used for non-alcoholic steatohepatitis, its efficacy and mechanisms in ALD are unclear. This study aimed to explore how SZSG exerts protective effects against alcohol-triggered liver injury and the underlying mechanisms.

**Methods:**

Both acute and chronic ALD mouse models were established to assess liver injury, fibrosis, inflammation, gut microbiota composition, and serum metabolomic profiles.

**Results:**

SZSG treatment markedly attenuated hepatic steatosis, fibrosis, and inflammation, as evidenced by reductions in lipid accumulation, collagen deposition, and pro-inflammatory cytokine levels. Concurrently, it reshaped the gut microbiota and restored metabolites dysregulated by alcohol exposure. These beneficial effects appear to involve modulation of the TGF-β/Smad4 signaling pathway and its downstream effectors, α-SMA and Collagen I.

**Conclusion:**

Collectively, our findings suggest that SZSG combats ALD by simultaneously modulating the TGF-β/Smad4 signaling axis and preserving gut-liver homeostasis, thereby representing a promising multi-target therapeutic candidate.

## 1 Introduction

Alcohol-associated liver disease (ALD) is among the most common preventable causes of liver-related morbidity and mortality worldwide (Li et al., 2020a). Its pathological spectrum spans simple steatosis, steatohepatitis, fibrosis, cirrhosis, and even hepatocellular carcinoma (Argemi et al., 2020). Global statistics indicate that excessive alcohol intake accounts for roughly 3 million deaths annually, accounting for 5.3% of all fatalities; in individuals aged 20–39 years, this figure rises to 13.5% (Sempokuya et al., 2022).

ALD is characterized by ongoing hepatic injury, an inflammatory response, and oxidative stress (Gao et al., 2024). Despite considerable advances in understanding its pathogenesis, liver fibrosis remains a crucial determinant of disease progression and long-term prognosis (Lackner and Tiniakos, 2019). A defining feature of advanced ALD is hepatic fibrosis, which results from excessive extracellular matrix (ECM) accumulation driven by activated hepatic stellate cells (HSCs) (Taru et al., 2024). The development of alcohol-associated liver fibrosis involves multiple factors: chronic alcohol intake strongly upregulates cytochrome P450 2E1 (CYP2E1) activity (Zhu et al., 2025a), leading to severe oxidative stress (Osna et al., 2022), which directly damages lipids, proteins and DNA within hepatocytes; the resulting products can also act as chemokines to activate HSCs (Ryu et al., 2023). Concurrently, alcohol stimulates Toll-like receptor 4 (TLR4) on Kupffer cells (KCs, the liver-resident macrophages), stimulating the downstream nuclear factor-κB (NF-κB) pathway and enhancing the release of pro-inflammatory cytokines including tumor necrosis factor-α (TNF-α), interleukin-1β (IL-1β), and interleukin-6 (IL-6) (Patel et al., 2021). These cytokines not only exacerbate hepatocyte damage but also act directly on HSCs. Once activated, HSCs produce abundant type I and type III collagen (Rodríguez-Rodríguez et al., 2021) and secrete tissue inhibitor of metalloproteinases, which inhibit ECM degradation, leading to the net deposition of collagen (Dawood et al., 2020; Song et al., 2024). In addition, alcohol-mediated gut microbial dysbiosis promotes the portal translocation of lipopolysaccharide (LPS) to the liver, where it binds to KC surface TLR4, thus serving as a major trigger of pro-inflammatory signaling (Hong et al., 2019). The interplay between intestinal dysbiosis and inflammatory cascades is a key amplifier of ALD progression (Zhang et al., 2017). Consequently, disruption of the ‘gut-liver axis’ is considered a key amplifier in the transition from steatosis to hepatitis and fibrosis (Arab et al., 2020; Glaser et al., 2020).

Notably, the progression of ALD is intimately linked to the activation of the TGF-β/Smad signaling pathway. TGF-β1 signals primarily through TGF-βRI and TGF-βRII, leading to phosphorylation of Smad2/3, which then binds to Smad4 and translocates to the nucleus to activate fibrotic gene expression (Zhang et al., 2024). Smad4, as the obligatory co-mediator, is indispensable for transducing TGF-β signals that drive HSC activation and ECM deposition (Gao et al., 2022). Importantly, both alcohol metabolism-induced oxidative stress and gut-derived LPS have been shown to converge on and potentiate this pathway, linking the gut-liver axis to fibrotic outcomes at the molecular level. However, current treatment options of ALD remain largely restricted to alcohol abstinence and supportive care, with no approved drugs available to reverse established fibrosis (Bataller et al., 2022). Available pharmacological interventions, such as aldehyde dehydrogenase inhibitors and opioid receptor antagonists, carry multiple undesirable effects, including drug dependence, nausea, cutaneous manifestations, dizziness, and leukopenia (Cheng et al., 2023). Therefore, there is an urgent need to identify novel therapeutic modalities that can interrupt the vicious cycle of inflammation, gut-liver axis dysfunction, and Smad4-mediated fibrotic signaling.

Traditional Chinese Medicine (TCM) offers numerous herbal formulas whose therapeutic value lies in the synergistic interplay of active ingredients, allowing them to address multifaceted pathological cascades (Silitonga et al., 2024; Li, 2020). Shenze Shugan capsule (SZSG) is a traditional Chinese medicine formula composed of 9 herbal ingredients, such as Shanzha (*Crataegi Fructus*), Zexie (*Alismatis Rhizoma*), Huangqi (*Astragali Radix*), Gegen (*Puerariae Lobatae Radix*), Danshen (*Salvia miltiorrhiza Bge*), Huzhang (*Reynoutria japonica Houtt.*), Juemingzi (*Cassiae Semen*), Dahuang (*Rhei Radix et Rhizoma*) and Chaihu (*Bupleuri Radix*). *Crataegi Fructus* is rich in flavonoids and organic acids; via enhancing the enzymatic activity of antioxidants (SOD, GSH-Px) while restraining HSC activation, it reduces collagen deposition and exhibits a certain degree of anti-fibrotic potential (Li et al., 2022; Wang et al., 2019). *Astragali Radix* contains astragalosides, while rhubarb contains emodin, both of which inhibit HSCs activation and ECM synthesis (Huang et al., 2024; HaoShang et al., 2023; Liang et al., 2021). *Puerarin*, a compound found in *Puerariae Lobatae Radix*, protects liver cells by accelerating alcohol metabolism through the activation of alcohol dehydrogenase (ADH) and aldehyde dehydrogenase (ALDH), scavenging free radicals, and reducing endoplasmic reticulum stress. Furthermore, *puerarin* inhibits the activation of HSCs and ameliorates alcoholic liver fibrosis. Extracts from *S. miltiorrhiza Bge* possess anti-inflammatory properties and can inhibit TNF-α-induced activation of NF-κB (Han et al., 2019); they can also prevent cirrhosis by maintaining antioxidant activity (Meim et al., 2019); furthermore, they can treat hepatocyte damage by promoting hepatocyte regeneration and improving hepatic blood circulation, and prevent liver fibrosis by downregulating Transforming Growth Factor-beta (TGF-β) (Cao et al., 2020). SZSG has been approved in China for the treatment of non-alcoholic steatohepatitis (NASH) (Huang et al., 2024), but its efficacy in ALD remains uncertain. Given the shared pathogenic mechanisms between NASH and ALD, it is reasonable that SZSG possesses the potential to mitigate alcohol-associated hepatic damage.

Consequently, this study was designed to determine the therapeutic action of SZSG on alcoholic liver damage and to elucidate its underlying mechanisms. By establishing acute and chronic ALD mouse models, and combining analyses of liver pathology and liver function parameters with data on the gut microbiota, serum metabolites and relevant proteomics, the therapeutic efficacy of SZSG was comprehensively evaluated. The results indicate that SZSG attenuates the course of liver fibrosis in association with modulation of the TGF-β/Smad4 signaling pathway, thereby effectively delaying the progression of ALD, providing scientific evidence for its potential application in treating ALD.

## 2 Materials and methods

### 2.1 Animals and treatments

Male C57BL/6 mice (specific pathogen free, 7–8 weeks old, 24–26 g body weight) were purchased from Beijing Huafukang Biotechnology Company Limited (license No.: SCXK (JING) 2024-0003). Mice were maintained under standardized environmental conditions, with ambient temperature set at 22 °C–24 °C, relative humidity kept at 45%–55%, and a 12- h light/dark cycle (light period from 7:00 a.m. to 7:00 p.m). All experimental procedures followed the International Guidelines for the Ethical Use of Animals and were approved by the Animal Ethics Committee of Jilin University (SY202403022, SY202406018). Mice received standard chow and water without restriction and were subjected to a 7-day acclimatization phase before experimental manipulation.

#### 2.1.1 Medicines preparation

SZSG capsules (Jilin Aodong Pharmaceutical Industry Group Yanji Co., Ltd., Yanji, China; National Drug Approval Number: Z20130022, Implementation Standard: YBZ00602013, Batch No. A24021, specification: 0.45 g/capsule) and silymarin (Sil; Tianjin Tasly Shengte Pharmaceutical Co., Ltd., Tianjin, China, H200400299, Batch No. 450801001, specification:35 mg/capsule) were used in this study. The 1,137.5 mg/kg dose of SZSG was chosen based on clinical dose conversion: the recommended human dose is 4.5 g/day for a 60 kg adult (approximately 75 mg/kg/day), and applying a 9.1× safety margin yielded 1,137.5 mg/kg. Similarly, the 63 mg/kg dose of Sil was derived from the clinical dose of 420 mg/day for a 60 kg adult (approximately 7 mg/kg/day), also with a 9.1× safety margin yielded 63.7 mg/kg. For administration, the contents of the capsules or Sil were dissolved in sterile saline to final concentrations of 113.75 mg/mL (for SZSG) and 6.3 mg/mL (for Sil), freshly prepared daily, with a fixed gavage volume of 10 mL/kg body weight.

#### 2.1.2 Acute ALD model established and SZSG administration

A total of 32 mice were randomly divided into four experimental groups (n = 8 each): Control (Ctrl), Alcohol, Alcohol + Sil, and Alcohol + SZSG groups. Except for the Ctrl mice, mice underwent intragastric administration of 42% (v/v) ethanol at a daily volume of 10 mL/kg for 14 consecutive days. An identical volume of water was provided to the Ctrl group. Throughout this period, the Alcohol + Sil and Alcohol + SZSG groups were also treated with 63.7 mg/kg Sil and 1,137.5 mg/kg SZSG daily, respectively (Figure 1A).

**FIGURE 1:**
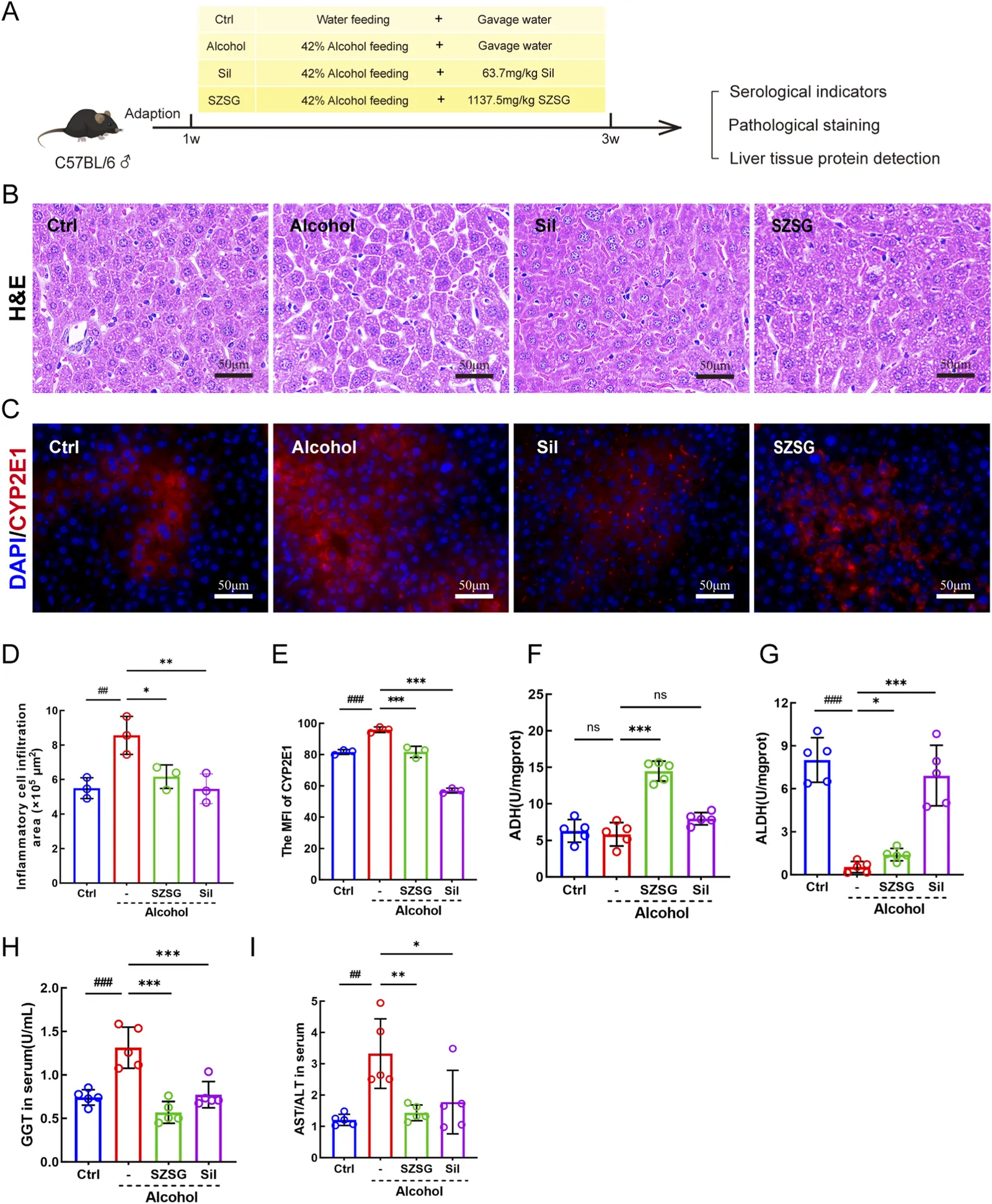
SZSG mitigates acute ALD in mice. Mice liver **(A)** Schematic diagram of animal experiments. **(B)** H&E staining (200 ×, scale bar: 50 μm) and **(C)** CYP2E1 IF staining (400 ×, scale bar: 50 μm). **(D)** Quantification of inflammatory cell infiltration area of liver H&E staining. **(E)** Quantification of the mean fluorescence intensity of liver CYP2E1 IF staining. Detection of **(F)** ADH, **(G)** ALDH in liver, **(H)** GGT levels, and **(I)** AST/ALT ratio in serum. ^##^ *P* < 0.01, ^###^ *P* < 0.001 vs. Ctrl mice; ^*^ *P* < 0.05, ^**^ *P* < 0.01, ^***^ *P* < 0.001 vs. Alcohol mice, n = 3 for **(B–E)**, n = 5 for **(F–I)**.

#### 2.1.3 Chronic ALD model established and SZSG administration

A total of 32 mice underwent randomization, producing the Ctrl group (8 mice) and the Alcohol group (24 mice). Chronic ALD was induced by stepwise increments of ethanol in the drinking water: 5% (v/v) during week 1, 10% (v/v) during week 2, and similarly progressive increases until a final 25% (v/v) concentration, which was maintained from week 5 through week 24. The ethanol solutions were freshly prepared and changed daily to minimize evaporation. Mice in the Ctrl group had unrestricted access to drinking water for 28 weeks. At the start of week 20, mice in Alcohol group were further divided into 3 groups (n = 8): the Alcohol group, the Alcohol + Sil group, and the Alcohol + SZSG group. From week 21 to week 28, 10 mL/kg saline, 63 mg/kg Sil, and 1,137.5 mg/kg SZSG were administered daily by gavage to the Alcohol mice, the Alcohol + Sil mice, and the Alcohol + SZSG mice, respectively (Figure 2A).

**FIGURE 2:**
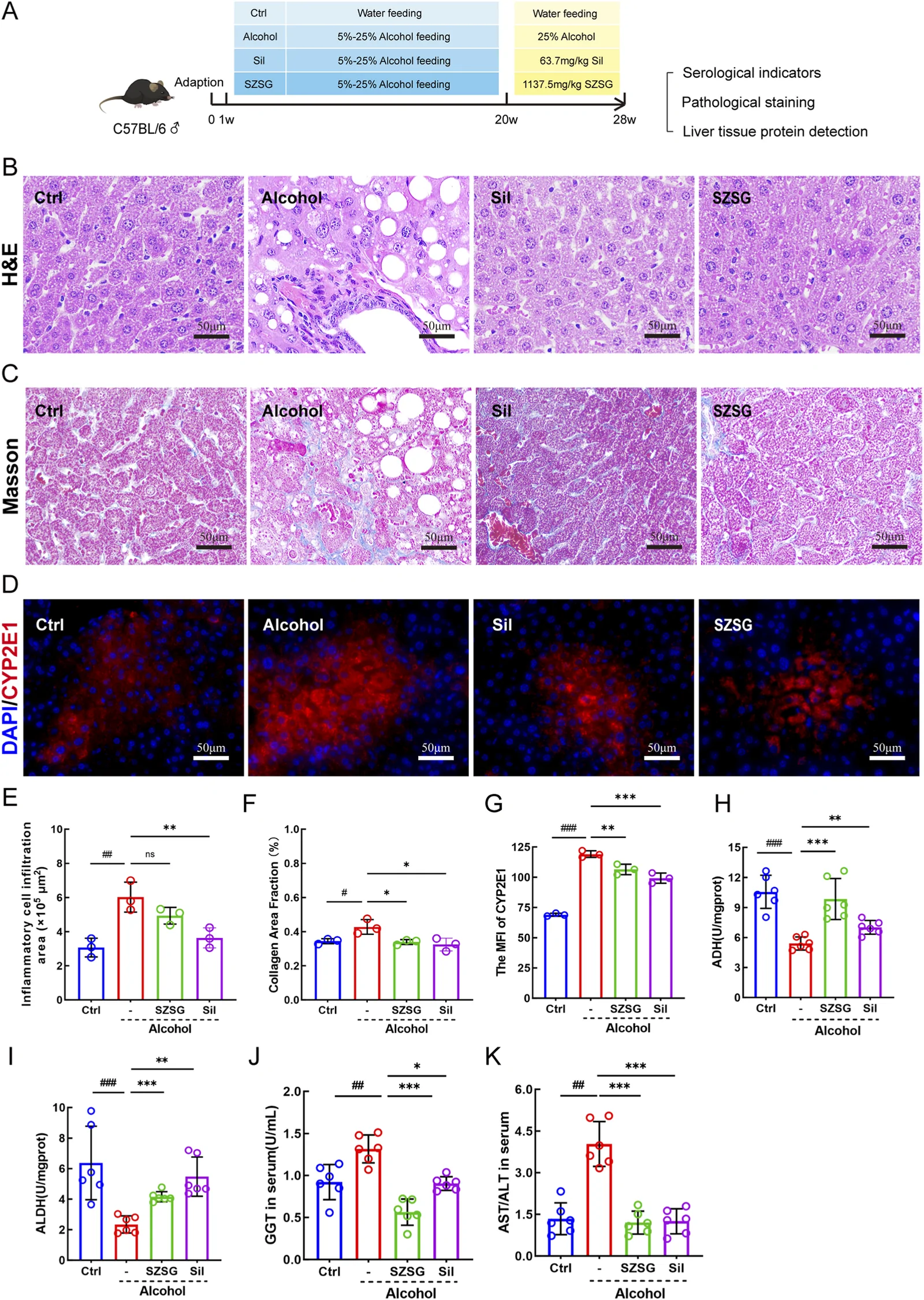
SZSG alleviates chronic ALD in mice. Mice liver **(A)** Schematic diagram of animal experiments. **(B)** H&E staining (200 ×, scale bar: 50 μm), **(C)** Masson staining (200 ×, scale bar 50 μm), and **(D)** CYP2E1 IF staining (400 × , scale bar 50 μm). **(E)** Quantification of inflammatory cell infiltration area of liver H&E staining. **(F)** Quantification of collagen area fraction of liver Masson staining. **(G)** Quantification of the mean fluorescence intensity of liver CYP2E1 IF staining. Detection of **(H)** ADH, **(I)** ALDH in the liver, **(J)** GGT levels, and **(K)** AST/ALT ratio in serum. ^#^ *P* < 0.05, ^##^ *P* < 0.01, ^###^ *P* < 0.001 vs. Ctrl mice; ^*^ *P* < 0.05, ^**^ *P* < 0.01, ^***^ *P* < 0.001 vs. Alcohol mice, n = 3 for **(B–G)**, n = 6 for **(H–K)**.

At the end of the experiment, all mice were fasted 12 h and euthanized by CO_2_ inhalation (70% v/v). Post-euthanasia, blood, cecal contents, and major organs (liver, spleen, kidneys, heart, lungs) were retrieved for subsequent evaluation.

### 2.2 Biochemical and immunological analysis

The quantities of aspartate aminotransferase (AST), alanine aminotransferase (ALT), gamma-glutamyl transferase (GGT) in serum, ADH and ALDH in liver were quantified with commercial biochemical kits, strictly adhering to the manufacturers’ specifications. Enzyme-linked immunosorbent assay (ELISA) kits were used to measure the concentrations of glutathione peroxidase (GSH-Px) in liver and interleukin-2 (IL-2), interleukin-10 (IL-10), interferon-γ (IFN-γ), TNF-α, superoxide dismutase (SOD), and alkaline phosphatase (ALP) in serum. Further details concerning the kits are presented in Supplementary Table S1.

### 2.3 Histopathological analysis

Liver samples were rapidly excised, immersed in 4% paraformaldehyde for 24 h, dehydrated, embedded in paraffin, and sliced into 4-μm serial sections. Sections were first deparaffinized in xylene and then rehydrated through a series of graded ethanols; subsequently, they were stained with hematoxylin and eosin (H&E) or Masson’s trichrome using kits (B1002 and B1006, Baiqiandu, Wuhan, China). Photomicrographs were obtained with an inverted microscope (DS-U3, Nikon, Tokyo, Japan).

### 2.4 Gut microbiota analysis

Cecal contents from the Ctrl, Alcohol, and Alcohol + SZSG mice (n = 5) were subjected to total genomic DNA extraction using the MagBeads FastDNA Kit for Soil (116564384, MP Biomedicals, Santa Ana, CA, United States), strictly adhering to the manufacturer’s instructions. Samples were selected for further sequencing based on the quantity and quality of extracted DNA. The V3-V4 hypervariable region of the 16 S rRNA gene was amplified with primers 799F (5′-AACMGGATTAGATACCCKG-3′) and 1193R (5′-ACG​TCA​TCC​CCA​CCT​TCC-3′). Subsequently, PCR products were purified and quantified before sequencing on the Illumina NovaSeq platform. The 16 S rRNA sequencing reads were subjected to denoising and outlier exclusion via QIIME2, then clustered into operational taxonomic units (OTUs) at a 97% similarity level. Subsequent data processing was carried out on the Paisennuo Cloud platform. Statistical analyses encompassed α-diversity, β-diversity, and tests for significant intergroup differences. Differentially abundant genera were identified using Linear Discriminant Analysis Effect Size (LEfSe) combined with the Wilcoxon rank-sum test, and a Benjamini-Hochberg false discovery rate (FDR) < 0.1 was considered statistically significant.

### 2.5 Untargeted metabolomics analysis

Serum samples from the Ctrl, Alcohol, and Alcohol + SZSG mice (n = 5) were first precipitated with methanol-acetonitrile, then centrifuged (14,000 g, 20 min) for non-targeted metabolomic analysis. The supernatants were analyzed using a high-performance liquid chromatography (HPLC) system (1,290, Agilent, Santa Clara, CA, United States) in tandem a mass spectrometer (TripleTOF 6600, SCIEX, Framingham, MA, United States). Metabolite identification was achieved by matching precise mass (<25 ppm) and MS/MS spectra against public databases. Data were processed using SIMCA-P 16.1 (Umetrics, Umea, Sweden). Differential metabolites were selected based on a projected variable importance in the projection (VIP) ≥ 1 and *P* < 0.05 (Student’s t-test). To control the false discovery rate, the Benjamini-Hochberg (BH) procedure was applied (FDR < 0.1). For pathway enrichment analysis, differentially expressed metabolites were mapped to the Kyoto Encyclopedia of Genes and Genomes (KEGG) database (https://www.kegg.jp/) using the R package clusterProfiler (v4.10.0). Pathway enrichment was considered significant when the impact value was >0.1 and the *P*-value (from hypergeometric test) was <0.05. The top 20 enriched pathways were visualized. An integrated analysis was performed using the differentially expressed bacterial genera and metabolites identified above. Spearman’s rank correlation coefficients were calculated to assess the associations between significant serum metabolites, and between key bacterial genera and significant serum metabolites. The R package pheatmap was used to visualise these correlations as heatmaps. All statistical tests were two-tailed; in the correlation analysis, a nominal *P*-value <0.05 was considered statistically significant.

### 2.6 Proteomics analysis

Similar to our previous studies (Zhang et al., 2025), proteins were extracted and quantified, precipitated with acetone, and subsequently treated with iodoacetamide. Peptide samples were collected after hydrolysis with trypsin (V5113, Promega Corporation, Madison, WI, United States). Peptide samples were desalted using a Peptide Desalting Spin Column (89851, Thermo Fisher Scientific, Waltham, MA, United States). Following peptide separation, gradient elution was performed using a high-performance liquid chromatography system (Vanquish Neo, Thermo Fisher Scientific) with a C18 column, followed by data-independent acquisition (DIA) analysis using a high-resolution mass spectrometer (Orbitrap Astral LC-MS, Thermo Fisher Scientific). All data analysis was based on a 99% confidence level for protein identification, with an FDR ≤1%. Differentially expressed proteins were identified with using a fold change (FC) ≥ 1.5, combined with a *P*-value <0.05 (Student’s t-test) and an FDR <0.1 (BH-adjusted). Perform KEGG enrichment analysis using the R package clusterProfiler (v4.10.0). Calculate the *P*-value using the hypergeometric test (with a significance threshold of *P* < 0.05) to identify significantly enriched functional entries, and thereby determine the primary biological functions of the differentially expressed proteins.

### 2.7 Western blot

Liver specimens (15–20 mg) were disrupted in ice-cold RIPA lysis buffer (1:15, w/v) that had been supplemented with 1% protease and phosphatase inhibitors. The homogenates were lysed in two cycles of 30 s each at 60 Hz using a tissue homogenizer (SCIENTZ-48, Ningbo Xinzhi Biotechnology Co., Ltd., Ningbo, China), and then centrifuged at 12,000 × g for 10 min at 4 °C. Protein concentration was assessed employing a BCA assay kit (A55864, Thermo Fisher Scientific). Following this, 30 µg of protein was separated via SDS-PAGE and subsequently transferred to a PVDF membrane. Subsequent to blocking, the membrane was incubated with primary antibody at 4 °C overnight, followed by a secondary antibody conjugated with HRP. Membranes were visualized using enhanced chemiluminescence (ECL) reagents and quantified with ImageJ software (Media Cybernetics, Rockville, MD, United States). A complete description of all antibodies is presented in Supplementary Table S2.

### 2.8 Immunofluorescence (IF) analysis

Following prior studies (Zhang et al., 2025), sections underwent blocking after dewaxing and antigen retrieval with EDTA (pH 9.0) (C1051, Baiqidu Biotechnology, Wuhan, China). Subsequently, primary and secondary antibodies were incubated sequentially at 4 °C. Cells were stained with DAPI for 10 min, mounted, and imaged using a fluorescence microscope. Detailed antibody information is summarized in Supplementary Table S2.

### 2.9 Statistic analysis

All data are expressed as mean ± standard error of the mean (S.E.M). Mice were randomly assigned to treatment groups, and investigators were blinded to the group allocation during histological assessments. Normality was assessed using the Shapiro-Wilk test, and homogeneity of variances was evaluated by Levene’s test. For data meeting parametric assumptions, one-way analysis of variance (ANOVA) followed by Tukey’s *post hoc* test was used for multi-group comparisons. For data that did not meet the assumptions, the Kruskal-Wallis test was used. Outliers were identified using Grubbs’ test (α = 0.05). For high-throughput omics data, the Benjamini-Hochberg (BH) procedure was applied to control the false discovery rate (FDR) at < 0.1. Statistical analyses were performed using GraphPad Prism 10.5 (GraphPad Software, San Diego, CA, United States), and a *P*-value <0.05 was considered statistically significant unless otherwise specified.

## 3 Results

### 3.1 SZSG ameliorated acute alcoholic liver injury

An acute ALD mice model was established over 2 weeks, with treatment administered as described to demonstrate the rapid hepatoprotective effect of SZSG against alcohol induced acute inflammation (Figure 1A). H&E staining revealed that 42% ethanol induced hepatic steatosis with inflammatory cell infiltration in the livers of acute ALD mice compared to the Ctrl mice, including structural disorganization, fatty vacuoles, and cellular swelling (Figure 1B). Following treatment with SZSG, the area of inflammatory cell infiltration was reduced by 27% (*P* < 0.01; Figure 1D). Immunofluorescence testing showed that SZSG significantly downregulated CYP2E1 protein expression by 13.79% (*P* < 0.01; Figures 1C,E) and upregulated ADH by 141.67% (*P* < 0.001; Figure 1F) and ALDH by 210.42% (*P* < 0.001; Figure 1G). SZSG intervention significantly suppressed the elevation of GGT in serum by 56.72% (*P* < 0.001, Figure 1H). Treatment with SZSG significantly inhibited the rise AST levels in serum (*P* < 0.05, Supplementary Figure S1B), no significant changes were observed in serum ALT levels (Supplementary Figure S1C), and the AST/ALT ratio was reduced by 22.06% (P < 0.01, Figure 1I). Concurrently, ALD mice exhibited significant weight loss (*P* < 0.001, Supplementary Table S3) accompanied by markedly elevated liver-to-body weight ratios (*P* < 0.001, Supplementary Table S4), no significant changes were observed following administration of SZSG. No pathological changes were observed in the heart, spleen, lungs or kidneys across all groups (Supplementary Figure S1A), suggesting that SZSG did not cause overt organ toxicity under the conditions of this study.

### 3.2 SZSG ameliorated chronic alcoholic liver injury

A chronic alcoholic liver injury mouse model was established over 28 weeks, with SZSG administered from weeks 21–28 to evaluate its anti-fibrotic efficacy and underlying mechanism (Figure 2A). Histopathological analysis revealed that SZSG treatment effectively mitigated alcohol-induced liver injury, manifested by reduced lipid droplet accumulation, suppressed neutrophil and inflammatory cell infiltration, decreased collagen fiber deposition, and restored hepatic architecture (Figures 2B,C). The area of inflammatory cell infiltration was reduced by 18% (*P* < 0.01; Figure 2E). The proportion of collagen area decreased by 24% (*P* < 0.05; Figure 2F). SZSG significantly downregulated CYP2E1 protein expression by 10.25% (*P* < 0.01; Figures 2D,G) and upregulated ADH by 90.57% and ALDH by 55.77% (*P* < 0.001; Figures 2H,I) in chronic ALD mice. SZSG significantly suppressed GGT levels in serum by 57.25% (*P* < 0.001; Figure 2J). Treatment with SZSG significantly increased ALT levels in serum (*P* < 0.05, Supplementary Figure S2C) (Supplementary Figure S2B), no significant changes were observed in serum AST levels (Supplementary Figure S2B), and the AST/ALT ratio was reduced by 65.85% (*P* < 0.001, Figure 2K). Consistent with the acute model, chronic alcohol exposure led to significant weight loss (*P* < 0.001; Supplementary Table S5) and increased liver-to-body weight ratios (*P* < 0.001; Supplementary Table S6), no significant changes were observed following administration of SZSG. No pathological changes were observed in other major organs (Supplementary Figure S2A), suggesting that SZSG did not cause overt organ toxicity under the conditions of this study.

### 3.3 SZSG modulated gut microbiota and serum metabolite levels in chronic ALD mice

Venn diagram analysis of the gut microbiota showed that the unique OTUs in the Ctrl, Alcohol and Alcohol + SZSG groups were 5413, 4,805 and 4,703, respectively (Figure 3A). Compared with the Alcohol groups, the SZSG group exhibited significantly lower alpha-diversity indices, including Simpson, Pielou_e, and Shannon (*P* < 0.05, Figure 3B). β-diversity evaluated by principal coordinate analysis (PCoA) indicated that SZSG treatment substantially modified the structural organization of the intestinal microbiota in chronic ALD mice (Figure 3C). SZSG treatment induced changes in 12 genera-level bacteria within the mouse gut microbiota, among which the following were significantly altered. Compared with ALD mice, *Ruminococcus* and *Coprococcus* showed significantly reduced, while *Allobaculum* and *Akkermansia* showed significant increases in SZSG treated ALD mice (*P* < 0.05, Figure 3D; Supplementary Table S7).

**FIGURE 3:**
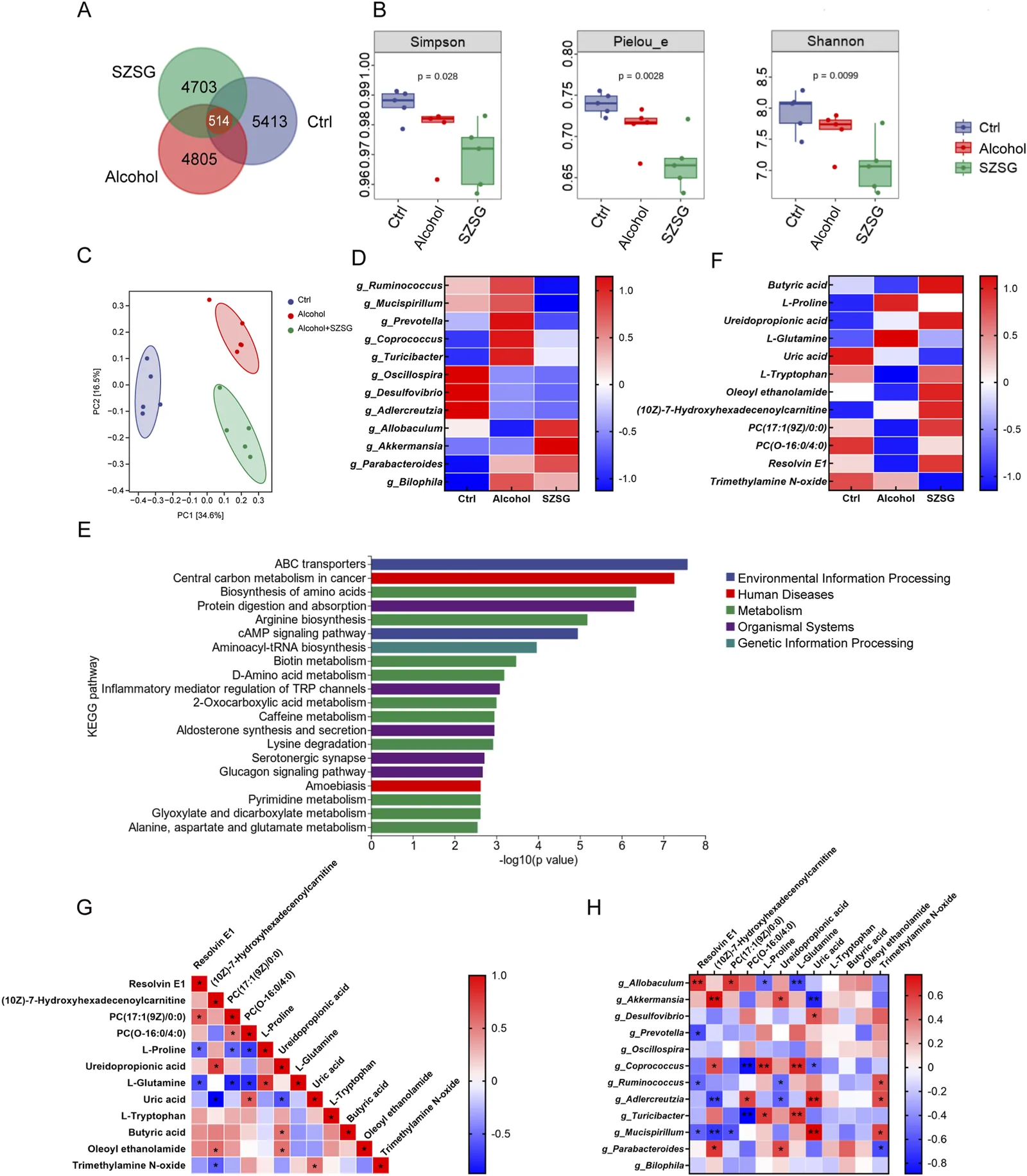
SZSG modulates gut microbiota and serum metabolite levels in ALD mice. **(A)** Venn diagram of gut microbiota. **(B)** Alpha diversity analysis of the microbial community. **(C)** PCoA analysis based on β-diversity. **(D)** Heatmap of the relative abundance of the top 12 microbial genera. **(E)** KEGG pathway analysis plot. **(F)** Heatmap of 12 differential metabolites. **(G)** Twelve differential metabolite correlation analysis plots. **(H)** Heatmap of interactions between the 12 differential gut microbes and 12 differential metabolites. n = 5, ^*^ *P* < 0.05, ^**^ *P* < 0.01.

LC-MS-based serum metabolomics was performed to identify metabolite changes. KEGG pathway enrichment analysis of the top 20 pathways revealed that SZSG-altered metabolites were mainly involved in human disease pathways (e.g., cancer, choline metabolism in alcoholism), body systems (e.g., bile secretion, cholesterol metabolism, regulation of lipolysis in adipocytes), and metabolism (e.g., glycerophospholipid metabolism, histidine metabolism) (Figure 3E). Heatmap analysis clearly illustrated the expression changes of 12 differentially representative metabolites between the Alcohol and Alcohol + SZSG groups showed that compared with the Alcohol mice, SZSG increased the levels of Butyric acid, L-Tryptophan, Oleoyl ethanolamide (OEA), PC(17:1 (9Z)/0:0), PC(O-16:0/4:0), and Resolvin E1, whereas the levels of L-Proline and Trimethylamine N-oxide (TMAO) were significantly lower (Figure 3F; Supplementary Table S8). And the correlation between these metabolites was analyzed by Pearson’s method, revealing positive correlations between OEA and ureidopropionic acid, and between TMAO and butyric acid. (VIP >1, *P* < 0.05) (Figure 3G; Supplementary Table S9). The relationship between the 12 differential bacterial genera and serum 12 differential metabolites were further investigated by Spearman’s analysis (VIP >1, *P* < 0.05). Spearman correlation analysis was performed to explore potential associations between microbial genera and serum metabolites. Correlation heatmaps showed that *Allobaculum* abundance was positively correlated with OEA, *Ruminococcus* abundance was positively correlated with TMAO and negatively correlated with OEA (Figure 3H; Supplementary Table S10).

### 3.4 SZSG regulated protein expression in the liver of chronic ALD mice

Proteomic analysis was employed to investigate the pathways through which SZSG exerts its alleviating effects on ALD. According to the Venn diagram, comparison between the Ctrl group and the Alcohol group identified 354 differentially expressed proteins, while comparison between the Alcohol group and the Alcohol + SZSG group revealed 156 differentially expressed proteins (Figure 4A). Heatmap analysis clearly illustrated the expression changes of 12 differentially expressed proteins between the Alcohol and Alcohol + SZSG groups. Following SZSG administration, Smad4 and Gsdma was significantly downregulated, while Pi3kcd, Pltp, Ebf1, Fam3a, H2-Aa, Stat1, Zdhhc13, Extl2, Cyp2j6 and Atp6v0d2 was upregulated, indicating a regulatory effect of SZSG on the protein expression profile (Figure 4B; Supplementary Table S11). There is a close link between the protein Smad4 and Stat1 (Figure 4C). Notably, KEGG enrichment analysis indicated that the differentially expressed proteins were involved in inflammation- and fibrosis-related pathways (Figure 4D). Smad4 is a key co-mediator in the TGF-β/Smad signaling pathway and a transcriptional mediator essential for TGF-β-driven activation of HSCs and ECM deposition (Wei et al., 2022). Stat1, on the other hand, acts as a negative regulator of TGF-β signaling by IFN-γ-induced phosphorylation of Stat1, which then promotes Smad7 expression, thereby antagonizing TGF-β signaling and inhibiting HSC activation (Guo et al., 2022). Smad4 and Stat1 represent, respectively, the pro-fibrotic and anti-fibrotic key nodes in the fibrotic signaling pathway, and there is a clear regulatory relationship between the two (Guo et al., 2009). Given the importance of these biological functions, we selected these two proteins as the focus of our study. Additionally, type I collagen alpha 1 chain (Collagen I) and alpha-smooth muscle actin (α-SMA) are the fibrosis markers in the chronic ALD mice (Liu et al., 2020). Western blot analysis confirmed that SZSG treatment significantly decreased Smad4 expression (*P* < 0.05; Figures 4E,F) and increased Stat1 phosphorylation (*P* < 0.01; Figures 4E,G) in chronic ALD mice liver. SZSG treatment suppressed the expression of Collagen I (*P* < 0.01; Figures 4E,H) and α-SMA (*P* < 0.001; Figures 4E,I). Immunofluorescence staining further confirmed the effects of SZSG on Collagen I (*P* < 0.01; Figures 4J,K) and α-SMA (*P* < 0.01; Figures 4L,M) expression.

**FIGURE 4:**
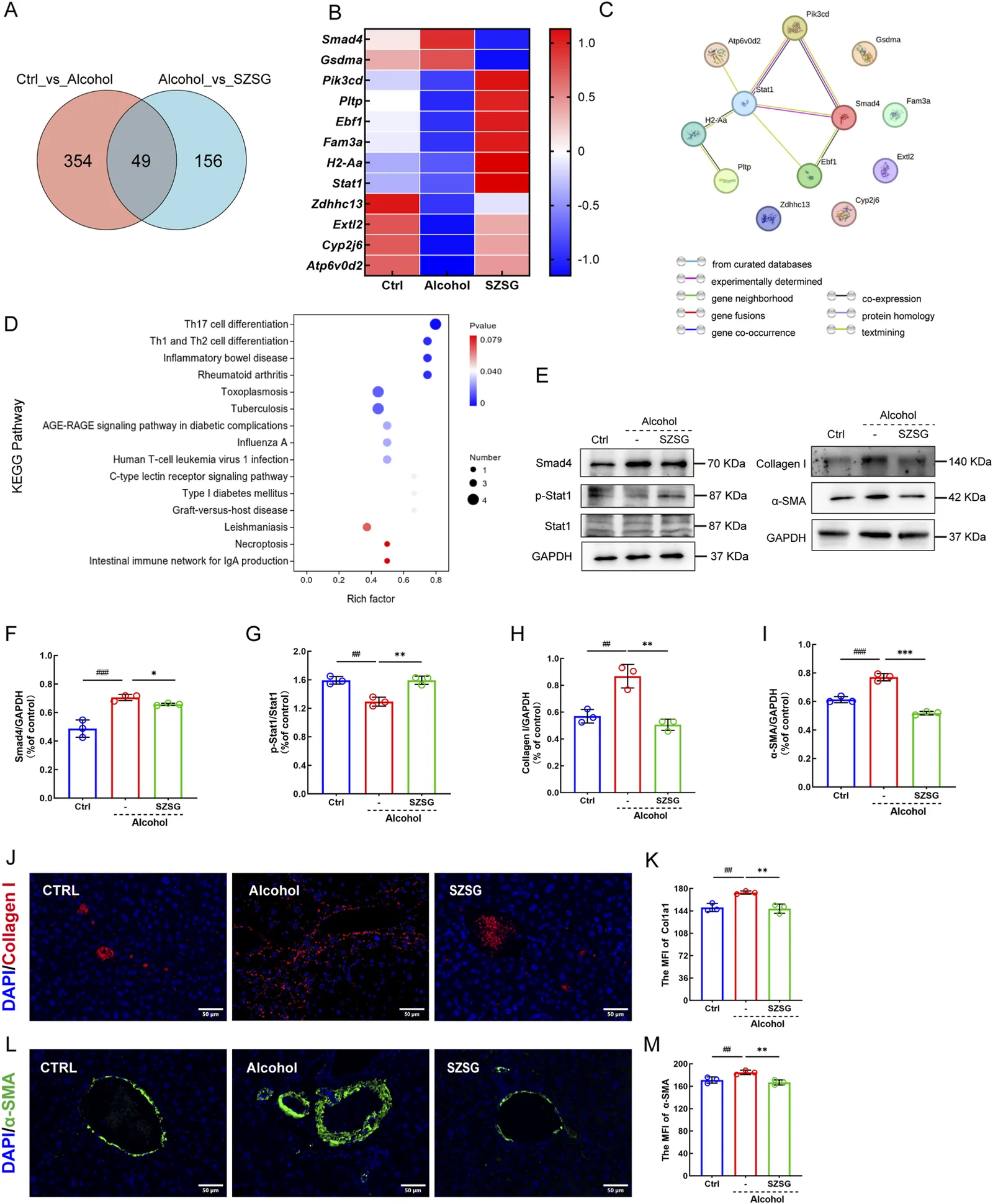
SZSG regulates protein expression in the livers of mice with chronic ALD. **(A)** Differential protein Venn chart. **(B)** Twelve differential protein heatmap. **(C)** Relationships among the 12 differentially expressed proteins. **(D)** KEGG enrichment analysis plot. **(E)** The expression of ALD-related differential proteins. Semi-quantitative analysis of proteins **(F)** Smad4, **(G)** p-Stat1/Stat1 **(H)** Collagen I and **(I)** α-SMA in **(E)** via ImageJ. IF detection of the liver **(J)** Collagen I and **(L)** α-SMA. Semi-quantitative analysis of **(K)** Collagen I and **(M)** α-SMA in **(J,L)** via ImageJ. Scale bar: 50 μm ^##^ *P* < 0.01, ^###^ *P* < 0.001 vs. Ctrl mice; ^*^ *P* < 0.05, ^**^ *P* < 0.01, ^***^ *P* < 0.001 vs. Alcohol mice, n = 5 for **(A–D)**, n = 3 for **(E–M)**.

### 3.5 SZSG-mediated inhibition of hepatic fibrosis in ALD mice is associated with the TGF-β/smad4 pathway

The pathophysiology of liver fibrosis begins with an inflammatory response to parenchymal cell injury, activating nonparenchymal and resident immune cells, releasing pro-inflammatory and pro-fibrotic mediators, which subsequently driving the activation of HSCs (TARU et al., 2024). Western blot analysis revealed that alcohol exposure significantly elevated the protein levels of TNF-α, IL-6, and IL-1β in the liver, whereas SZSG treatment reversed these changes (*P* < 0.05, Figures 5A–D). Furthermore, SZSG treatment significantly reduced the expression of TGF-β, TGF-β receptor (TGF-βRII) and p-Smad2/3 (*P* < 0.05, Figures 5A,E–G), while increased Smad7 expression (*P* < 0.01, Figures 5A,H). ELISA results showed that, compared with the Alcohol mice, SZSG had significantly increased serum levels of IL-2, IL-10, IFN-γ, SOD and hepatic level of GSH-Px, and decreased TNF-α and ALP levels (Figures 5I–O).

**FIGURE 5:**
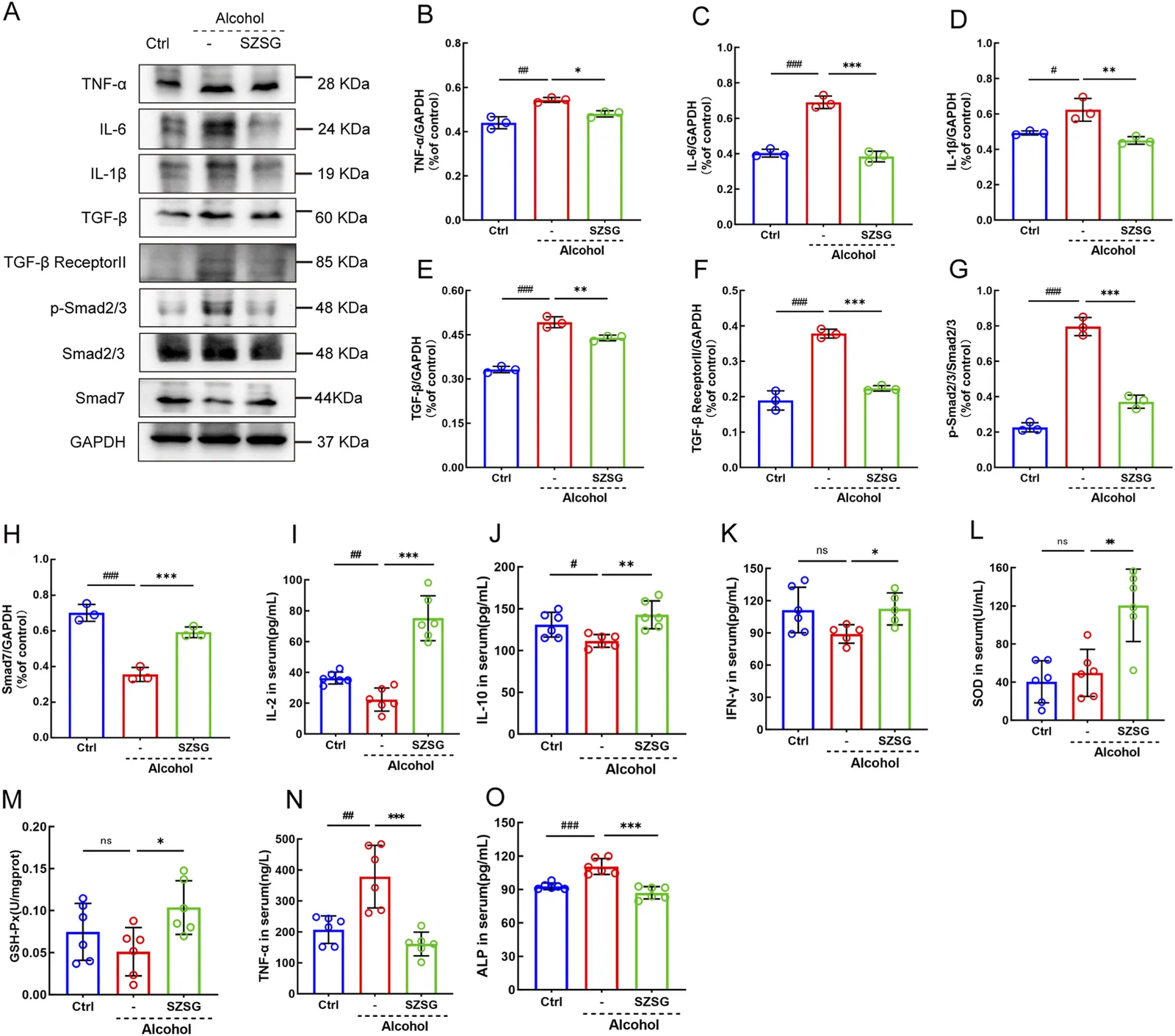
SZSG inhibits liver fibrosis in ALD mice through the TGF-β1/Smad4 pathways. **(A)** The expression of TGF-β1/Smad4 pathway proteins in liver tissue. Semi-quantitative analysis of proteins **(B)** TNF-α, **(C)** IL-6, **(D)** IL-1β, **(E)** TGF-β, **(F)** TGF-β ReceptorII, **(G)** p-Smad2/3, **(H)** Smad7 proteins in **(A)** via ImageJ. SZSG treatment increased the levels of **(I)** IL-2, **(J)** IL-10, **(K)** IFN-γ, **(L)** SOD in serum and **(M)** GSH-Px in liver, reduced the levels of **(N)** TNF-α and **(O)** ALP in serum of chronic ALD mice. ^#^ *P* < 0.05, ^##^ *P* < 0.01, ^###^ *P* < 0.001 vs. Ctrl mice; ^*^ *P* < 0.05, ^**^ *P* < 0.01, ^***^ *P* < 0.001 vs. Alcohol mice, n-3 for **(A–H)**, n = 6 for **(I–O)**.

## 4 Discussion

As a growing global health concern, ALD is featured by a complex network of interactions encompassing hepatic inflammation, metabolic dysfunction, gut dysbiosis, and HSC activation (Bouare, 2022). Current therapies to reverse established fibrosis remain very limited (Gao et al., 2024; Wu et al., 2023). Traditional Chinese medicine formulas, capable of orchestrating complex pathological networks through synergistic interactions among their bioactive ingredients, represent a promising therapeutic modality for ALD (Wu et al., 2025; Dong et al., 2025). The present investigation systematically established the therapeutic efficacy of SZSG against alcohol-related hepatic injury and dissected its hierarchical mechanisms. Our findings indicate that SZSG exerts its protective effects by promoting ethanol metabolism at the metabolic level; improving the gut microbiome and metabolome at the intestinal level; and attenuating the TGF-β/Smad4 pro-fibrotic signaling cascade at the hepatic level. This multi-level regulatory mechanism makes SZSG a highly attractive therapeutic candidate for the treatment of ALD. The sequential processing of ethanol by ADH and ALDH constitutes the primary pathway of hepatic alcohol metabolism, whereby ethanol is first oxidized to acetaldehyde, then to acetate, with carbon dioxide and water as end products (Robinson and Shah, 2020; Contreras-Zentella et al., 2022; Yan et al., 2023). Acetaldehyde, the intermediate metabolite, is the core toxic molecule inducing liver damage by interfering with DNA replication, causing DNA damage, and forming adducts (Jiang et al., 2020). Efficient metabolism of ethanol and acetaldehyde constitutes a vital defense mechanism against alcohol-induced liver toxicity. CYP2E1 serves as a key enzyme involved in alcohol metabolism. Chronic alcohol consumption induces CYP2E1 activation, leading to the production of toxic metabolites and reactive oxygen species (Carrasco et al., 2021). GGT is involved in the degradation of extracellular glutathione, providing precursors for intracellular antioxidant synthesis to counteract oxidative stress induced by alcohol metabolism. However, this process also generates reactive oxygen species, and elevated GGT levels may reflect the extent of hepatocyte injury (Mitrić and Castellano, 2023; Corti et al., 2020; Pomacu et al., 2022). In our study, SZSG treatment significantly upregulated the expression of ADH and ALDH while downregulating the expression of CYP2E1, suggesting that SZSG accelerates the clearance of ethanol and its toxic intermediate metabolite, acetaldehyde. This metabolic shift not only alleviates direct hepatotoxic damage but also suppresses the production of reactive oxygen species (ROS) and acetaldehyde-protein adducts (known to trigger HSC activation and TGF-β signaling) (Kurniawan et al., 2020; Ceni et al., 2014), thereby intervening in ethanol metabolism at the earliest stage of the disease process.

Consistent with previous studies (Yan et al., 2023), chronic alcohol exposure in our mouse model resulted in significant hepatic steatosis, inflammation, and collagen deposition. SZSG treatment markedly downregulated CYP2E1 protein expression while upregulating ADH and ALDH, key alcohol metabolism enzymes. SZSG treatment also significantly decreased serum GGT and AST/ALT levels and improved hepatic histopathology. These results confirmed the pronounced hepatoprotective effects of SZSG against ALD. These hepatoprotective effects are comparable to those reported for other bioactive compounds derived from natural foods (Zhou et al., 2025), highlighting the therapeutic potential of multi-component natural products in combating metabolic and inflammatory disturbances associated with alcoholic liver disease (ALD). Moreover, no adverse histological changes were observed in major organs following SZSG administration.

Emerging evidence underscores that dysregulation of the gut–liver axis is a critical driver in the pathogenesis of ALD (Albillos et al., 2020). Chronic alcohol consumption disrupts gut microbial homeostasis and impairs intestinal barrier function, thereby promoting the translocation of bacterial endotoxins, particularly lipopolysaccharide (LPS), into the portal circulation (Meroni et al., 2019; Raya Tonetti et al., 2024). Once in the liver, LPS activates Kupffer cells through Toll-like receptor 4 (TLR4), initiating pro-inflammatory cascades and ultimately contributing to hepatic stellate cell (HSC) activation and fibrogenesis (Raya Tonetti et al., 2024; Balakrishnan et al., 2025). In the present study, 16 S rRNA sequencing and untargeted metabolomics jointly revealed that SZSG profoundly reshaped both the gut microbial community and its associated metabolic landscape in alcohol-fed mice. Specifically, SZSG enriched beneficial taxa such as *Akkermansia* and *Allobaculum*, which are well recognized for reinforcing intestinal epithelial integrity and reducing systemic endotoxemia (Koppinger et al., 2020; Wu et al., 2020; Zhao et al., 2024), while simultaneously suppressing the expansion of *Ruminococcus*, a pro-inflammatory genus linked to heightened intestinal permeability and elevated LPS production (Bai et al., 2024; Tan et al., 2025).

Complementing these microbial shifts, our metabolomic analysis identified that SZSG modulated key metabolites with established hepatic bioactivities. Notably, SZSG administration significantly increased OEA, an endogenous lipid mediator known to directly inhibit HSC activation (Chen et al., 2015), and decreased TMAO, a metabolite that promotes lipid accumulation, inflammatory responses, and HSC activation (Zhou et al., 2022). Importantly, correlation analyses further strengthened the microbe–metabolite interplay: *Allobaculum* abundance positively correlated with OEA levels, whereas *Ruminococcus* abundance showed a positive correlation with TMAO and a negative correlation with OEA. These integrative data suggest that SZSG not only restores a favorable gut ecology but also redirects metabolic outputs that jointly alleviate hepatic inflammation and fibrotic signaling. Collectively, our findings support that SZSG ameliorates chronic alcohol-induced liver injury through a coordinated action on the gut–liver axis, enhancing protective microbial populations, reducing endotoxin-related stimuli, and adjusting metabolite profiles that directly influence HSC activity, thereby offering a multi-targeted therapeutic strategy for ALD.

The functional significance of this gut-centric mechanism extends beyond intestinal barrier preservation. By reducing the portal influx of LPS and other microbial products, SZSG indirectly attenuates TLR4-mediated activation of Kupffer cells, thereby diminishing the hepatic inflammatory milieu that primes HSCs for activation. Importantly, LPS has been shown to sensitize HSCs to TGF-β stimulation by downregulating the inhibitory TGF-β receptor BAMBI (a pseudoreceptor that dampens TGF-β signaling), effectively lowering the threshold for TGF-β/Smad4-driven fibrogenesis (Chen et al., 2024). Furthermore, gut microbiota imbalance can directly exacerbate alcohol-induced liver fibrosis through the TGF-β/Smad signaling pathway (; Zhou et al., 2019). Crosstalk between TLR4 signaling and TGF-β signaling in HSCs has been well documented, highlighting the role of TLR signaling in stellate cell activation and the progression of liver fibrosis (Li et al., 2020b; Liu et al., 2021). Thus, the gut restorative effects of SZSG may not be confined to the intestine but instead exert profound indirect effects on hepatic fibrosis via the gut–liver axis.

Liver fibrosis is a complex process involving inflammation, HSCs activation and ECM deposition (Wang et al., 2022). TGF-β1 signaling pathway plays a central role in fibrosis. TGF-β1 signals primarily through TGF-βRI and TGF-βRII, leading to phosphorylation of Smad2/3, which then binds to Smad4 and translocates to the nucleus to activate fibrotic gene expression (Chen et al., 2025). Targeted inhibition of the TGF-β1 pathway effectively alleviates the fibrotic process (Zhu et al., 2025b). Smad7 acts as an endogenous inhibitor of TGF-β/Smad signaling by antagonizing TGF-β1 function (Xu et al., 2023). Additionally, IFN-γ promotes Smad7 expression via Stat1 phosphorylation, thereby counteracting TGF-β signaling and HSC activation (Guo et al., 2009). The TGF-β/Smad4 signaling cascade serves as a primary regulator of HSCs activation and ECM production in liver fibrosis (Chen et al., 2025).

In this study, SZSG treatment significantly reduced the expression of TGF-β1, TGF-βRII, Smad4 and Smad2/3 phosphorylation, while increased Smad7 level and Stat1 phosphorylation. These changes were accompanied by decreased expression of collagen I and α-SMA, key markers of fibrosis. These findings indicate that SZSG may exert its anti-fibrotic effects at least partly by modulating the TGF-β/Smad4 signaling axis, consistent with previous reports that Smad4 deficiency attenuates fibrosis (Wei et al., 2022). Furthermore, SZSG treatment reduced pro-inflammatory cytokines (IL-1β, TNF-α, and IL-6) and restored anti-inflammatory cytokines (IL-2, IL-10, and IFN-γ). The observed increase in GSH-Px and SOD activity, along with decreased ALP levels, further indicates that SZSG enhances antioxidant capacity and improves hepatocyte function, thereby mitigating alcohol-induced liver injury. SZSG improves ALD by accelerating ethanol clearance, improving the intestinal barrier to limit LPS translocation, and inhibiting TGF-β/Smad4 signaling. This comprehensive, multi-pronged strategy distinguishes SZSG from traditional single-target drugs, which are unable to address the multifactorial nature of ALD.

Several limitations of this study should be acknowledged. First, the single dose, male-only mice, and lack of daily ethanol/food/water intake and blood ethanol monitoring limit real-world evidence and translational value. Second, proteomic and Western blot data suggest TGF-β/Smad4 involvement, but the omics analyses (n = 5) and microbiota–metabolite correlations remain exploratory; targeted multi-omics and functional interventions are needed to confirm pathway dependency. Third, owing to the proprietary nature of the patented SZSG formulation, and the current lack of consensus on representative quality markers for this complex nine-ingredient Chinese herbal preparation, this study was unable to conduct a comprehensive chemical fingerprint analysis or a complete quantitative analysis of all major constituents. Future research should establish comprehensive chemical fingerprint profiles and identify candidate bioactive biomarkers, thereby enabling more reliable quality control and inter-batch reproducibility in subsequent preclinical and clinical studies.

## 5 Conclusion

In summary, this study indicates that SZSG effectively alleviates alcohol-induced acute and chronic liver injury. The underlying mechanisms involve modulation of the TGF-β/Smad4 signaling, inhibition of HSCs activation, restoration of gut microbiota homeostasis, and regulation of key metabolites. These findings provide a mechanistic basis for clinical evaluation of SZSG in ALD and support broader investigation of multi-targeted traditional Chinese medicine strategies in chronic fibrotic liver diseases.

## Data Availability

The raw 16S rRNA gene amplicon sequencing data reported in this paper have been deposited in the Genome Sequence Archive (GSA) at the National Genomics Data Center (NGDC), China National Center for Bioinformation (CNCB), under accession number CRA048482, and are publicly accessible at https://ngdc.cncb.ac.cn/gsa. The mass spectrometry proteomics data have been deposited to the ProteomeXchange Consortium via the iProX partner repository (https://www.iprox.cn) with the dataset identifier PXD082579. All other data generated or analyzed during this study are included in this published article and its Supplementary Material files.
