# Supplementary material for: Shenze shugan capsules alleviate alcoholic liver injury in association with modulation of the TGF-β/smad4 signaling pathway
Source: Front Pharmacol. 2026 Oct 2;17:1942947. doi: 10.3389/fphar.2026.1942947 (PMC13671995; doi:10.3389/fphar.2026.1942947)
Supplement: Supplementary file 1 [file Supplementaryfile1.docx]

**Supplement Figures**

**Fig. S1**. **Safety Evaluation of Acute Alcoholic Liver Injury in Mice.** (A) H&E staining of heart, spleen, lung and kidney. (200 ×, scale bar: 50 μm). (B) AST and (C) ALT levels in serum. ^#^ *P* < 0.01 *vs*. Ctrl mice; ^*^ *P* < 0.05, ^**^ *P* < 0.01 *vs.* Alcohol mice, n = 5 for B-C.

**Fig. S2**. **Safety Evaluation of Chronic Alcoholic Liver Injury in Mice.** (A) H&E staining of heart, spleen, lung and kidney. (200 ×, scale bar: 50 μm). (B) AST and (C) ALT levels in serum. ^#^ *P* < 0.01 *vs*. Ctrl mice; ^*^ *P* < 0.05 *vs.* Alcohol mice, n = 5 for B-C.

**Supplementary Tables**

**Table S1.** Details of cytokines ELISA kits.

| **Cytokines** | **Catalog number** | **Company** | **Area** |
| --- | --- | --- | --- |
| AST | C010-2-1 | Nanjing Jiancheng Bioengineering Institute | Nanjing, China |
| ALT | C009-2-1 | Nanjing Jiancheng Bioengineering Institute | Nanjing, China |
| ADH | A083-2-1 | Nanjing Jiancheng Bioengineering Institute | Nanjing, China |
| ALDH | A075-1-1 | Nanjing Jiancheng Bioengineering Institute | Nanjing, China |
| GTT | C017-2-1 | Nanjing Jiancheng Bioengineering Institute | Nanjing, China |
| IL-2 | CK-E20010M | Shanghai Shuoxin Biotechnology Co., Ltd. | Shanghai, China |
| IL-10 | CK-E20005M | Shanghai Shuoxin Biotechnology Co., Ltd. | Shanghai, China |
| IFN-γ | CK-E11382M | Shanghai Shuoxin Biotechnology Co., Ltd. | Shanghai, China |
| TNF-α | CK-E20220M | Shanghai Shuoxin Biotechnology Co., Ltd. | Shanghai, China |
| GSH-Px | KT2755-A | Nanjing Kote Biotechnology Co., Ltd. | Nanjing, China |
| SOD | KT2389-A | Nanjing Kote Biotechnology Co., Ltd. | Nanjing, China |
| ALP | CK-E20105M | Shanghai Shuoxin Biotechnology Co., Ltd. | Shanghai, China |

**Table S2**. Details of all antibodies in the article.

| **Antibody** | **Molecular weight** | **Catalog number** | **Dilution for Western blot** | **Company** | **Area** |
| --- | --- | --- | --- | --- | --- |
| Collagen I | 140 kDA | A16891 | 1:1000 | Abclonal | Wuhan, China |
| FITC-labelled goat anti-rabbit | / | C4101 | 1:500 | Baiqiandu | Wuhan, China |
| FITC-labelled goat anti-mouse | / | C4102 | 1:500 | Baiqiandu | Wuhan, China |
| α-SMA | 42 kDA | 19245s | 1:1000 | Cell Signaling | Boston, USA |
| Smad4 | 70 kDa | A19116 | 1:1000 | Abclonal | Wuhan, China |
| Stat1 | 87 kDa | A0027 | 1:2000 | Abclonal | Wuhan, China |
| pStat1 | 87 kDa | AP0054 | 1:1000 | Abclonal | Wuhan, China |
| Smad7 | 44 kDa | A12343 | 1:1000 | Abclonal | Wuhan, China |
| TGF-β | 60 kDa | 3709T | 1:1000 | Cell Signaling | Boston, USA |
| TGF-β ReceptorII | 85 kDa | 79424T | 1:1000 | Cell Signaling | Boston, USA |
| Smad2/3 | 48 kDa | 5339T | 1:1000 | Cell Signaling | Boston, USA |
| pSmad2/3 | 48 kDa | 8828T | 1:1000 | Cell Signaling | Boston, USA |
| TNF-α | 28kDa | A24214 | 1:2000 | Abclonal | Wuhan, China |
| IL-6 | 24 kDa | A0286 | 1:1000 | Abclonal | Wuhan, China |
| IL-1β | 19 kDa | A22257 | 1:2000 | Abclonal | Wuhan, China |
| GAPDH | 37 kDA | E-AB-48016 | 1:2000 | Elabscience | Wuhan, China |
| Goat-Anti-Mouse | / | E-AB-1001 | 1:2000 | Elabscience | Wuhan, China |
| Goat-Anti-Rabbit | / | E-AB-1003 | 1:2000 | Elabscience | Wuhan, China |

**Table S3.** The effects of SZSG on body weight (g) of acute alcohol-induced mice.

|  | **Ctrl** | **Alcohol** | **Sil** | **SZSG** |
| --- | --- | --- | --- | --- |
| 1^st^ day | 23.45±0.23 | 23.66±0.23 | 23.84±0.26 | 23.90±0.34 |
| 3^rd^ day | 24.20±0.39 | 21.80±0.41^###^ | 21.91±0.37 | 21.85±0.57 |
| 5^th^ day | 24.21±0.42 | 19.56±1.11^###^ | 20.94±0.46^**^ | 20.58±1.21 |
| 7^th^ day | 23.31±0.69 | 20.05±0.37^###^ | 20.78±0.29^**^ | 21.13±0.78^**^ |
| 9^th^ day | 23.74±0.40 | 21.40±0.37^###^ | 21.85±0.17^*^ | 22.40±0.44^***^ |
| 11^th^ day | 24.27±0.38 | 21.88±0.28^###^ | 21.86±0.34 | 22.05±0.75 |
| 12^th^ day | 24.15±0.37 | 21.69±0.63^###^ | 22.45±0.45^*^ | 22.39±1.44 |
| 13^th^ day | 24.23±0.44 | 21.90±0.30^###^ | 22.36±0.45^*^ | 22.10±1.57 |
| 14^th^ day | 23.35±0.31 | 20.68±0.40^###^ | 21.30±0.39^*^ | 21.21±1.32 |

The data were analyzed using a one-way ANOVA and expressed as means ± SEM, ^###^*P* < 0.001 *vs.* Ctrl mice, ^*^*P* < 0.05, ^**^*P* < 0.01 and ^***^*P* < 0.001 *vs.* vehicle-treated alcohol-administered mice.

**Table S4.** The effects of SZSG on organ indices (mg/g) of acute alcohol-induced mice.

|  | **Ctrl** | **Alcohol** | **Sil** | **SZSG** |
| --- | --- | --- | --- | --- |
| Liver index | 39.59±2.14 | 50.78±3.10^###^ | 42.42±3.23^***^ | 46.51±2.49^*^ |
| Heart index | 7.50±1.53 | 6.17±0.66 | 6.65±1.10 | 6.23±0.74 |
| Spleen index | 2.41±0.23 | 2.52±0.26 | 2.37±0.26 | 2.37±0.19 |
| Kidney index | 12.57±0.82 | 14.47±2.05^#^ | 12.71±0.90 | 12.72±0.37^*^ |
| Lung index | 6.69±0.50 | 7.57±0.94^#^ | 8.16±1.36 | 7.42±0.92 |

The data were analyzed using a one-way ANOVA and expressed as means ± SEM, ^#^*P* < 0.05 and ^###^ *P* < 0.001 *vs.* Ctrl mice, ^*^*P* < 0.05 and ^***^ *P* < 0.001 *vs.* vehicle-treated alcohol-administered mice.

**Table S5.** The effects of SZSG on body weight (g) of chronic alcohol-induced mice.

|  | **Ctrl** | **Alcohol** | **Sil** | **SZSG** |
| --- | --- | --- | --- | --- |
| 1^st^ week | 21.91±0.50 | 20.15±0.48^###^ | 20.50±0.23 | 20.70±0.64 |
| 3^rd^ week | 24.19±0.69 | 19.93±3.06^##^ | 22.28±0.37 | 22.06±0.92 |
| 6^th^ week | 27.41±0.67 | 21.16±2.67^###^ | 23.49±1.14 | 22.75±1.79 |
| 9^th^ week | 27.20±1.34 | 22.49±2.37^###^ | 23.64±0.33 | 23.79±0.58 |
| 12^th^ week | 27.66±2.58 | 23.74±1.87^##^ | 25.30±1.28 | 25.26±1.50 |
| 15^th^ week | 28.78±2.30 | 19.95±3.42^###^ | 23.00±1.92 | 22.89±2.06 |
| 18^th^ week | 29.81±1.82 | 20.61±1.59^###^ | 22.73±1.70^*^ | 22.80±1.33^*^ |
| 20^th^ week | 30.31±1.82 | 21.59±1.03^###^ | 23.04±0.99^*^ | 22.75±1.20 |
| 23^th^ week | 29.69±2.31 | 21.73±1.63^###^ | 21.78±0.84 | 22.03±0.97 |
| 26^th^ week | 30.53±2.11 | 21.61±1.98^###^ | 23.56±0.85^*^ | 23.74±1.31^*^ |
| 28^th^ week | 29.44±1.66 | 21.78±3.37^###^ | 23.41±1.22 | 22.45±1.71 |

The data were analyzed using a one-way ANOVA and expressed as means ± SEM, ^##^ *P* < 0.01 and ^###^ *P* < 0.001 *vs.* Ctrl mice, ^*^*P* < 0.05 *vs.* vehicle-treated alcohol-administered mice.

**Table S6.** The effects of SZSG on organ indices (mg/g) of chronic alcohol-induced mice.

|  | **Ctrl** | **Model** | **Sil** | **SZSG** |
| --- | --- | --- | --- | --- |
| Liver index | 36.08±1.83 | 47.78±1.60^###^ | 44.51±2.07^**^ | 43.98±2.50^**^ |
| Heart index | 6.90±0.98 | 6.86±1.44 | 5.68±0.43 | 6.01±1.04 |
| Spleen index | 2.55±0.42 | 1.19±0.39^###^ | 1.99±0.37^**^ | 1.80±0.41^*^ |
| Kidney index | 11.67±1.35 | 15.61±1.08^###^ | 15.67±1.08 | 14.31±0.98^*^ |
| Lung index | 7.15±1.39 | 7.21±1.21 | 8.00±2.05 | 6.92±0.93 |

The data were analyzed using a one-way ANOVA and expressed as means ± SEM, ^###^ *P* < 0.001 *vs.* Ctrl mice, ^*^*P* < 0.05 and ^**^ *P* < 0.01 *vs.* vehicle-treated alcohol-administered mice.

| **Taxa** | **Mean of Ctrl** | **Mean of Alcohol** | **Mean of SZSG** | **Ctrl vs Alcohol** | **Alcohol vs SZSG** |
| --- | --- | --- | --- | --- | --- |
|  |  |  |  | ***P*-value** | ***P*-value** |
| *Ruminococcus* | 0.037362 | 0.049116 | 0.009193 | 0.323555 | 0.004369 |
| *Mucispirillum* | 0.007048 | 0.008872 | 0.000427 | 0.742303 | 0.129074 |
| *Prevotella* | 0.056601 | 0.104231 | 0.04066 | 0.22225 | 0.086295 |
| *Coprococcus* | 0.011532 | 0.096189 | 0.042129 | 0.001234 | 0.020055 |
| *Turicibacter* | 0.000415 | 0.014577 | 0.006486 | 0.000436 | 0.029616 |
| *Oscillospira* | 0.074659 | 0.047425 | 0.045049 | 0.168991 | 0.893681 |
| *Desulfovibrio* | 0.123583 | 0.052365 | 0.044051 | 0.033351 | 0.696961 |
| *Adlercreutzia* | 0.034665 | 0.008175 | 0.005954 | 0.0048 | 0.316353 |
| *Allobaculum* | 0.377576 | 0.251283 | 0.475497 | 0.166253 | 0.023462 |
| *Akkermansia* | 0.000383 | 0.004706 | 0.151588 | 0.045987 | 0.017116 |
| *Parabacteroides* | 0.00354 | 0.005681 | 0.006398 | 0.128573 | 0.63976 |
| *Bilophila* | 0.001872 | 0.002443 | 0.002315 | 0.703701 | 0.937688 |

**Table S7.** Relative abundances of the top 12 genus in the CTRL, Alcohol and SZSG

Data are presented as the mean. Ctrl: Ctrl mice; Alcohol: vehicle-treated alcohol-administered mice; SZSG: SZSG-treated alcohol-administered mice.

**Table S8.** The differential metabolites of serum among Ctrl, Alcohol and SZSG.

| **Metabolites** | **Mean of Ctrl** | **Mean of Alcohol** | **Mean of SZSG** | **Ctrl vs Alcohol** | | **Alcohol vs SZSG** | |
| --- | --- | --- | --- | --- | --- | --- | --- |
|  |  |  |  | **VIP** | ***P*-value** | **VIP** | ***P*-value** |
| Resolvin E1 | 794678.4844 | 683894.2008 | 866477.7333 | 1.488916 | 0.004969 | 1.020883 | 0.034752 |
| (10Z)-7-Hydroxyhexadecenoylcarnitine | 2936177.475 | 5312979.688 | 9021122.51 | 1.493689 | 0.001785 | 1.616616 | 0.001667 |
| PC(17:1(9Z)/0:0) | 46866258.44 | 38782905.96 | 51860900.66 | 1.343319 | 0.008789 | 1.763330 | 0.000219 |
| PC(O-16:0/4:0) | 14557593.62 | 2355685.623 | 7215844.167 | 1.579517 | 0.000017 | 1.736353 | 0.000341 |
| L-Proline | 35162702.52 | 41070668.32 | 38012199 | 1.269977 | 0.016151 | 1.33913 | 0.021590 |
| Ureidopropionic acid | 2118661.426 | 2705315.211 | 3707352.818 | 0.865493 | 0.125444 | 1.289256 | 0.030021 |
| L-Glutamine | 16581533.06 | 26260288.67 | 18194294.97 | 1.394833 | 0.010483 | 1.859271 | 0.000000 |
| Uric acid | 16747174.12 | 11941688.44 | 9514536.573 | 1.428639 | 0.000974 | 1.303435 | 0.028594 |
| L-Tryptophan | 964198257.6 | 855254255.7 | 980493099.4 | 0.854151 | 0.118183 | 1.362291 | 0.036474 |
| Butyric acid | 985990.7028 | 958510.4828 | 1038596.025 | 0.699098 | 0.233482 | 1.378919 | 0.014618 |
| Oleoyl ethanolamide | 15794834.29 | 8247722.751 | 30621778.32 | 0.960520 | 0.073297 | 1.403869 | 0.016632 |
| Trimethylamine N-oxide | 3964393.073 | 2768863.178 | 898499.2284 | 0.490400 | 0.401679 | 1.531056 | 0.010681 |

Data are presented as the mean. Differences were considered statistically significant at *P* < 0.05 and VIP > 1. Ctrl: Ctrl mice; Alcohol: vehicle-treated alcohol-administered mice; SZSG: SZSG-treated alcohol-administered mice.

**Table S9.** Spearman correlation coefficient of differential metabolites.

| **ρ** | **Resolvin E1** | **(10Z)-7-Hydroxyhexadecenoylcarnitine** | **PC(17:1(9Z)/0:0)** | **PC(O-16:0/4:0)** | **L-Proline** | **Ureidopropionic acid** | **L-Glutamine** | **Uric acid** | **L-Tryptophan** | **Butyric acid** | **Oleoyl ethanolamide** | **Trimethylamine N-oxide** |
| --- | --- | --- | --- | --- | --- | --- | --- | --- | --- | --- | --- | --- |
| **Resolvin E1** | 0 | 0.277089 | 0.006113 | 0.335204 | 0.044098 | 0.510299 | 0.011675 | 0.379613 | 0.179352 | 0.107313 | 0.083953 | 0.192372 |
| **(10Z)-7-Hydroxyhexadecenoylcarnitine** | 0.277089 | 0 | 0.201189 | 0.093438 | 0.23866 | 0.000449 | 0.979861 | 1.98E-05 | 0.75466 | 0.063238 | 0.022883 | 0.02931 |
| **PC(17:1(9Z)/0:0)** | 0.006113 | 0.201189 | 0 | 0.036524 | 0.036903 | 0.43498 | 0.000598 | 0.542608 | 0.263314 | 0.105256 | 0.155055 | 0.306736 |
| **PC(O-16:0/4:0)** | 0.335204 | 0.093438 | 0.036524 | 0 | 0.004953 | 0.247491 | 0.001093 | 0.010395 | 0.212362 | 0.559276 | 0.962018 | 0.159515 |
| **L-Proline** | 0.044098 | 0.23866 | 0.036903 | 0.004953 | 0 | 0.145917 | 0.000124 | 0.213083 | 0.618224 | 0.664142 | 0.828428 | 0.790793 |
| **Ureidopropionic acid** | 0.510299 | 0.000449 | 0.43498 | 0.247491 | 0.145917 | 0 | 0.749326 | 0.017437 | 0.210642 | 0.029137 | 0.033359 | 0.213636 |
| **L-Glutamine** | 0.011675 | 0.979861 | 0.000598 | 0.001093 | 0.000124 | 0.749326 | 0 | 0.500309 | 0.275867 | 0.180399 | 0.188514 | 0.568087 |
| **Uric acid** | 0.379613 | 1.98E-05 | 0.542608 | 0.010395 | 0.213083 | 0.017437 | 0.500309 | 0 | 0.427317 | 0.320793 | 0.205159 | 0.032787 |
| **L-Tryptophan** | 0.179352 | 0.75466 | 0.263314 | 0.212362 | 0.618224 | 0.210642 | 0.275867 | 0.427317 | 0 | 0.091617 | 0.137108 | 0.848147 |
| **Butyric acid** | 0.107313 | 0.063238 | 0.105256 | 0.559276 | 0.664142 | 0.029137 | 0.180399 | 0.320793 | 0.091617 | 0 | 0.506591 | 0.597944 |
| **Oleoyl ethanolamide** | 0.083953 | 0.022883 | 0.155055 | 0.962018 | 0.828428 | 0.033359 | 0.188514 | 0.205159 | 0.137108 | 0.506591 | 0 | 0.135082 |
| **Trimethylamine N-oxide** | 0.192372 | 0.02931 | 0.306736 | 0.159515 | 0.790793 | 0.213636 | 0.568087 | 0.032787 | 0.848147 | 0.597944 | 0.135082 | 0 |

**Table S10.** Spearman correlation coefficient between gut microbiota and differential metabolites.

| **ρ** | **Resolvin E1** | **(10Z)-7-Hydroxyhexadecenoylcarnitine** | **PC(17:1(9Z)/0:0)** | **PC(O-16:0/4:0)** | **L-Proline** | **Ureidopropionic acid** | **L-Glutamine** | **Uric acid** | **L-Tryptophan** | **Butyric acid** | **Oleoyl ethanolamide** | **Trimethylamine N-oxide** |
| --- | --- | --- | --- | --- | --- | --- | --- | --- | --- | --- | --- | --- |
| ***g_Allobaculum*** | 0.653571 | 0.146429 | 0.621429 | 0.45 | -0.52857 | 0.3 | -0.66071 | -0.21786 | 0.007143 | 0.303571 | 0.360714 | -0.41071 |
| ***g_Akkermansia*** | 0.214423 | 0.756787 | 0.212621 | -0.50813 | 0.243253 | 0.549572 | 0.219829 | -0.78742 | 0.111716 | 0.169376 | 0.050452 | -0.50092 |
| ***g_Desulfovibrio*** | -0.13571 | -0.43571 | -0.21786 | 0.396429 | -0.05 | -0.26786 | -0.12857 | 0.535714 | 0.292857 | -0.16429 | 0.25 | 0.482143 |
| ***g_Prevotella*** | -0.63929 | -0.12857 | -0.33571 | -0.26786 | 0.364286 | 0 | 0.460714 | 0.142857 | 0.035714 | 0.032143 | -0.50357 | 0.317857 |
| ***g_Oscillospira*** | -0.16429 | -0.23929 | -0.01071 | 0.292857 | -0.24286 | -0.325 | -0.43929 | 0.285714 | 0.028571 | 0.121429 | 0.125 | 0.232143 |
| ***g_Coprococcus*** | -0.45714 | 0.55 | -0.48929 | -0.87143 | 0.678571 | 0.285714 | 0.678571 | -0.55 | -0.375 | -0.03929 | -0.44643 | -0.2 |
| ***g_Ruminococcus*** | -0.54286 | -0.36786 | -0.32143 | -0.02857 | 0.2 | -0.52857 | 0.2 | 0.353571 | -0.05 | -0.30714 | -0.23929 | 0.535714 |
| ***g_Adlercreutzia*** | -0.25357 | -0.66786 | -0.11071 | 0.582143 | -0.26071 | -0.55357 | -0.20714 | 0.703571 | 0.15 | -0.025 | -0.01786 | 0.542857 |
| ***g_Turicibacter*** | -0.26273 | 0.421805 | -0.49687 | -0.88829 | 0.61126 | 0.312779 | 0.647007 | -0.45576 | -0.26452 | -0.20197 | -0.27346 | -0.2824 |
| ***g_Mucispirillum*** | -0.55357 | -0.71071 | -0.61071 | 0.007143 | 0.153571 | -0.43571 | 0.260714 | 0.775 | -0.21071 | -0.46429 | -0.23571 | 0.578571 |
| ***g_Parabacteroides*** | 0.039286 | 0.621429 | -0.14286 | -0.50714 | 0.171429 | 0.564286 | 0.110714 | -0.49643 | 0.207143 | 0.475 | 0.153571 | -0.63571 |
| ***g_Bilophila*** | -0.17143 | 0.128571 | -0.33571 | -0.31429 | 0.010714 | 0.1 | -0.125 | -0.125 | -0.19643 | 0.196429 | 0.014286 | -0.175 |

**Table S11.** The differential proteins among Ctrl, Alcohol, and SZSG.

| **Gene.Name** | **Proteins** | **Mean of Ctrl** | **Mean of Alcohol** | **Mean of SZSG** | **Ctrl vs Alcohol** | **Alcohol vs SZSG** |
| --- | --- | --- | --- | --- | --- | --- |
|  |  |  |  |  | ***P*-value** | ***P*-value** |
| Smad4 | P97471 | 25493.5 | 30440.6 | 18877.0333 | 0.18798672 | 0.01484374 |
| Gsdma | Q9EST1 | 268398.333 | 343025.139 | 90.6185 | 0.30592108 | 0.03075717 |
| Pik3cd | O35904 | 1223.45396 | 90.6185 | 3439.69707 | 0.00879489 | 0.01014383 |
| Pltp | P55065 | 11827.8 | 8488.98667 | 15120.7333 | 0.18023287 | 0.0280233 |
| Ebf1 | Q07802 | 8168.39837 | 6079.89 | 10722.8994 | 0.09319523 | 0.02548562 |
| Fam3a | Q9D8T0 | 12516.7 | 10194.8967 | 15557.4333 | 0.1029549 | 0.03810616 |
| H2-Aa | P14434 | 10471.12 | 6558.76667 | 25395.8667 | 0.20413755 | 0.04317789 |
| Stat1 | P42225 | 111670.7 | 82198.1333 | 215642.333 | 0.28278826 | 0.00313206 |
| Zdhhc13 | Q9CWU2 | 5243.63718 | 90.6185 | 2159.45564 | 0.01200809 | 0.00697848 |
| Extl2 | Q9ES89 | 4849.95665 | 90.6185 | 3863.07794 | 0.02746286 | 0.00202926 |
| Cyp2j6 | O54750 | 3171.88369 | 90.6185 | 2648.694 | 0.03996998 | 0.01478484 |
| Atp6v0d2 | Q80SY3 | 6794.23385 | 90.6185 | 5900.94268 | 0.04069158 | 0.04247422 |

Data are presented as the mean. Differences were considered statistically significant at *P* < 0.05. Ctrl: Ctrl mice; Alcohol: vehicle-treated alcohol-administered mice; SZSG: SZSG-treated alcohol-administered mice.
